# Bisphenol A impairs contractile function and vascular development in human cardiac organoids: Insights from toxicity assessment and evidence-based risk evaluation

**DOI:** 10.7555/JBR.39.20250533

**Published:** 2026-07-28

**Authors:** Linfeng Jiang, Yachang Wang, Huina Li, Yang Yang

**Affiliations:** 1 State Key Laboratory of Reproductive Medicine and Offspring Health, Nanjing Medical University, Nanjing, Jiangsu 211166, China; 2 Jiangsu Environmental Health Risk Assessment Engineering Research Center, Key Laboratory of Modern Toxicology of Ministry of Education, Center for Global Health, Nanjing Medical University, Nanjing, Jiangsu 211166, China

**Keywords:** cardiac organoids, bisphenol A, toxicity assessment, myocardium, endothelial cells

## Abstract

Bisphenol A (BPA), a widely encountered environmental endocrine-disrupting chemical, has been linked to an increased risk of congenital heart defects in epidemiological and experimental studies. To investigate its impact in a human-relevant system, we used a human cardiac organoid (hCO) model derived from human embryonic stem cells and exposed hCOs to BPA concentrations ranging from environmentally relevant to higher experimental levels (0.1–10 μmol/L) throughout differentiation. BPA exposure impaired cardiac contractile function and disrupted endothelial network formation, with more pronounced functional effects at higher concentrations. BPA exposure also induced apoptosis in hCOs. Subsequently, transcriptomic profiling revealed dysregulation of genes associated with cardiac rhythm and developmental signaling in BPA-treated hCOs. Among the concentrations tested, 0.5 μmol/L was the lowest concentration associated with a significant reduction in CD31 fluorescence intensity. This research provides *in vitro* evidence that BPA can perturb key processes in early human heart development, highlighting the necessity for further mechanistic and *in vivo* studies to clarify exposure relevance and its potential implications for fetal heart health.

## Introduction

Congenital heart defects (CHDs) are defined as structural malformations of the heart or great vessels present at birth. They are the most prevalent birth defects globally, affecting about 1% of live births^[1]^. Their etiology involves genetic factors such as aneuploidy and pathogenic variants, as well as environmental exposures^[2]^. Among environmental risk factors, endocrine-disrupting chemicals (EDCs) have garnered increasing attention. BPA, a typical EDC, is widely used in consumer products, including food containers, flame retardants, medical devices, and blood transfusion bags^[^^3^^–^^4^^]^. As a result, it has become a ubiquitous environmental contaminant, detectable in groundwater, rivers, lakes, marine systems, and agricultural soils^[5]^. The primary route of human exposure is dietary intake, followed by inhalation of dust. Crucially, BPA can penetrate the fetal environment. Lee *et al*^[^^6^^]^ quantified BPA levels in various human body fluids (serum, urine, placenta, breast milk, and cord serum), revealing a distribution pattern of neonatal urine > maternal urine > cord serum > maternal serum > breast milk > placenta. This pattern further demonstrates that BPA can cross the placental barrier to reach the fetus. Given that epidemiological studies have linked maternal BPA exposure to an increased risk of CHDs in offspring^[^^7^^]^, there is a critical need to elucidate its developmental cardiotoxicity.

Accumulating evidence from animal studies supports the adverse effects of BPA exposure on fetal heart development. In mice, both low- and high-dose BPA exposure induced cardiac abnormalities, including myocardial hyperemia, disorganized cardiomyocyte arrangement, and nuclear condensation with irregular chromatin distribution^[^^7^^]^. Similarly, studies in rat models have shown that gestational BPA exposure leads to increased collagen deposition in the ventricular septum^[8]^ and disrupts cardiomyocyte differentiation, promoting an aberrant shift toward a skeletal muscle-like phenotype, accompanied by DNA damage and reduced histone acetylation^[^^9^^]^. In zebrafish, BPA and its metabolite 4-methyl-2,4-bis(p-hydroxyphenyl)pent-1-ene interfere with estrogen-responsive elements, altering the expression of genes critical for valve formation and resulting in atrioventricular valve malformations, reduced cardiac contraction, and diminished blood flow^[^^10^^]^. These impairments in morphology and function mirror epidemiological data, thereby supporting the connection between maternal BPA exposure and a higher risk of offspring CHDs^[^^11^^]^, potentially mediated through epigenetic mechanisms that may also confer transgenerational susceptibility to cardiovascular disorders^[^^12^^]^.

To further delineate human-relevant effects, researchers have used human pluripotent stem cell-derived cardiomyocytes (hPSC-CMs) to examine BPA-induced developmental cardiotoxicity. Studies using this model have revealed several functional impairments induced by BPA exposure. Notably, Hyun *et al*^[^^13^^]^ reported that while BPA did not affect cell viability, it reduced action potential amplitude and contraction force in hPSC-CMs at higher concentrations. Similarly, Ma *et al*^[^^14^^]^ found that even low-dose BPA delayed cardiomyocyte repolarization, increasing the risk of arrhythmia. Chronic low-dose BPA/analog exposure triggered contractile dysfunction, Ca^2+^ dysregulation, and hypertrophy in hPSC-CMs^[^^15^^]^. Together, these studies indicate that BPA perturbs cardiac development and function across multiple species and model systems.

However, each of the current experimental models has inherent limitations that restrict their translational relevance for human developmental cardiotoxicity. Animal models, while informative, exhibit species-specific differences in cardiac development, electrophysiology, and xenobiotic metabolism that complicate extrapolation to humans. Two-dimensional (2D) hPSC-CM cultures offer a human cellular context but lack the complex three-dimensional (3D) architecture, extracellular matrix (ECM) organization, and cell-cell interactions of the developing heart. They also do not fully recapitulate the diversity of cardiac cell lineages (*e.g.*, endothelial cells [ECs] and fibroblasts) or the spatial patterning that influences cardiotoxic responses. Thus, there is a need for more physiologically relevant, human-based systems that better model the fetal heart to assess BPA-induced developmental cardiotoxicity.

Self-assembling human cardiac organoids (hCOs) from human embryonic stem cells (hESCs) represent a promising platform to address this gap. These hCOs, which include major cardiac cell types, recapitulate essential aspects of heart development^[^^16^^]^, such as spontaneous contraction and vascular network formation. This model has been successfully applied to study cardiac hypertrophy^[^^17^^]^ and CHDs induced by gestational diabetes^[^^16^^]^, highlighting its utility for modeling complex cardiac pathophysiology in a human context.

In the current study, we employed an hESC-derived hCO model to systematically investigate the developmental cardiotoxicity of BPA. Specifically, we aimed to assess its effects on contractile function, vascular development, and global gene expression, and to integrate these findings within the Targeted Risk Assessment of Environmental Chemicals (TRAEC) framework to evaluate the potential risk of BPA exposure to human fetal heart development. By combining a human 3D cardiac organoid system with quantitative functional and molecular readouts, this work seeks to provide a more mechanistically informed and human-relevant basis for evaluating BPA-associated CHD risk.

## Materials and methods

### Chemicals

BPA (molecular formula: C_15_H_16_O_2_, purity ≥ 99.5%, CAS No. 80-05-7) was purchased from Sinopharm Chemical Reagent Co., Ltd. (Sinopharm, Shanghai, China). A 100 mmol/L stock solution was prepared in cell culture-grade dimethyl sulfoxide (DMSO) and sterilized by passing it through a syringe filter. The aliquots were stored at 4 ℃ for subsequent experiments. Detailed information on all EDCs is provided in ***Supplementary Table 1***.

### Cell culture

H9 cells were cultured in Essential 8 Medium (Thermo Fisher Scientific, Waltham, MA, USA) on 12-well plates (NEST Biotechnology, Wuxi, China), pre-coated with Matrigel (Corning, NY, USA), at 37 ℃ in a humidified 5% CO_2_ incubator. Upon reaching 80%–90% confluency following daily medium changes, cells were passaged or prepared for hCO differentiation by dissociation with EDTA (Thermo Fisher Scientific).

### hCO formation

hCOs were differentiated from H9 cells as described previously^[16]^. Briefly, two days before differentiation (day −2), cells were digested with Accutase (FCMACS, Nanjing, China) and resuspended in Essential 8 Medium with 10 μmol/L Y27632 (Biochempartner, Shanghai, China). A total of 10000 cells in 100 μL were seeded per well in U-bottom ultra-low attachment 96-well plates (JET Biofil, Guangzhou, China) to form aggregates. On day −1, half of the medium was carefully replaced with 200 μL of fresh Essential 8 Medium without Y27632. Differentiation was initiated on day 0 by replacing the medium with 166 μL of RPMI/B-27 minus insulin containing CHIR99021 (4 μmol/L; Biochempartner), bone morphogenetic protein 4 (BMP4; 1.25 ng/mL; Stemimmune, San Francisco, CA, USA), and Activin A (1 ng/mL; Stemimmune). The medium was changed to plain RPMI/B-27 minus insulin on day 1. From day 2 to 3, the aggregates were treated with 2 μmol/L WNT-C59 (APExBIO, Shanghai, China). Fresh RPMI/B-27 minus insulin medium was replenished on days 4 and 6. On day 7, a 1-h pulse of 2 μmol/L CHIR99021 was applied, and the medium was subsequently switched to RPMI 1640 supplemented with 2% standard B-27 Supplement (Thermo Fisher Scientific). Thereafter, the medium was changed every other day until harvest on day 16.

### Toxicity assessment

We evaluated BPA concentrations ranging from 0.1 to 10 μmol/L, with 1 and 10 μmol/L used for the primary functional analyses, 1 μmol/L used for transcriptomic analysis, and lower concentrations (0.1–0.5 μmol/L) subsequently examined to characterize endothelial sensitivity. BPA treatment was initiated on differentiation day 0 and maintained continuously throughout the differentiation process by adding it to the culture medium during each change.

### RNA extraction and reverse transcription-quantitative PCR

Total RNA was extracted from hCOs on day 16 using TransZol Up reagent (TransGen Biotech, Beijing, China). All procedures were performed according to the manufacturer's protocol. RNA concentration and quality were determined using a NanoDrop 2000 spectrophotometer (Thermo Fisher Scientific). Reverse transcription was performed with 1 μg of total RNA using the One-Step gDNA Removal kit (TransGen Biotech). Quantitative PCR (qPCR) was carried out with ChamQ SYBR Color qPCR Master Mix (Low ROX Premixed; Vazyme, Nanjing, China) on a QuantStudio 7 Flex Real-Time PCR System (Applied Biosystems, Waltham, MA, USA). The thermal cycling conditions were as follows: pre-denaturation at 95 ℃ for 30 s; 40 cycles of 95 ℃ for 10 s and 60 ℃ for 30 s; and a melt-curve stage of 95 ℃ for 15 s, 60 ℃ for 60 s, and 95 ℃ for 15 s. All steps were performed according to the respective manufacturers' instructions. The sequences of primers used are listed in ***Supplementary Table 2***.

### Immunofluorescence (IF) staining

For IF staining, hCOs were first fixed in 4% paraformaldehyde (Ding Guo Chang Sheng, Beijing, China) for 1 h at room temperature. After washing, permeabilization and blocking were performed simultaneously by incubating the samples overnight at 4 ℃ in PBS buffer supplemented with 2.5% donkey serum (Jackson ImmunoResearch, West Grove, PA, USA) and 0.2% Triton X-100 (Beyotime, Shanghai, China). Primary antibody incubation was carried out overnight at 4 ℃, followed by PBS washes and incubation with fluorescent secondary antibodies. Finally, nuclei were stained with DAPI (YiFeiXue Biotechnology, Nanjing, China). See ***Supplementary Table 3*** for antibody specifications.

### Apoptosis analysis

Apoptosis was assessed using an Annexin Ⅴ-FITC/PI Apoptosis Detection Kit (Yeasen Biotechnology, Shanghai, China). Following collection on differentiation day 16, hCOs were washed with PBS and subsequently stained in a mixture containing 2.5 μmol/L Annexin Ⅴ-FITC, 5 μmol/L PI, and 1 μL Hoechst 33342 at 37 ℃ for 15–30 min. After staining, samples were washed twice with PBS and imaged using a fluorescence microscope.

### Evaluation of hCO morphology

Mean immunofluorescence intensity and organoid area were analyzed using ImageJ software (v2.0.0, NIH, Bethesda, MD, USA).

### RNA sequencing (RNA-seq) and data preprocessing

RNA-seq libraries were constructed following the SMART-seq2 protocol^[^^18^^]^. Briefly, samples were lysed, reverse-transcribed, and cDNA was amplified. Libraries were prepared *via* tagmentation, quality-checked, and sequenced on an Illumina NovaSeq 6000 platform (PE150; Illumina, San Diego, CA, USA). For data analysis, raw reads were first processed using Flexbar (v2.5) for adapter trimming and quality filtering. Cleaned reads were then aligned to the hg38 genome using STAR (v2.7.10b). Gene-level raw counts were generated using HTSeq (v0.13.5) and used as input for DESeq2 (v1.30.1) for differential expression analysis. For visualization and dimensionality reduction, transcripts per million (TPM) values were calculated from the raw counts to normalize for both sequencing depth and gene length, and PCA was performed on the log_2_(TPM + 1) transformed data.

### Differential expression and principal component analysis (PCA)

Following quantification, differential expression analysis was performed. Low-count genes (total counts < 10) were filtered out, and differentially expressed genes (DEGs) were identified using DESeq2 (v1.30.1) with a significance threshold of adjusted *P* < 0.05 and |log_2_(fold change [FC])| > 1. Functional enrichment of DEGs for Gene Ontology (GO) terms and Kyoto Encyclopedia of Genes and Genomes (KEGG) pathways was assessed using clusterProfiler (v4.8.3). To capture subtle coordinated changes, gene set enrichment analysis (GSEA) was also conducted against the GO/KEGG databases using a gene list ranked by signed −lg(*P* value) × sign(log_2_FC), with a false discovery rate (FDR) < 0.25 considered significant^[^^19^^]^. For dimensionality reduction and visualization, variance-stabilizing transformation (VST) was applied to the raw count data using DESeq2's vst function, which stabilizes variance across the mean expression range and is recommended for PCA of count-based RNA-seq data^[20]^. PCA was then performed on the VST-transformed values using the prcomp function in R (version 4.5.1), with centering and scaling applied.

### TRAEC strategy assessment process

#### Evidence collection

We conducted a systematic assessment of existing evidence on each chemical, including epidemiological, *in vivo*, and *in vitro* studies. Data from these three evidence streams were extracted and integrated for subsequent analysis.

#### Evidence scoring and conclusion

Each study was evaluated using a standardized multidimensional scoring system^[^^21^^]^.

**Reliability score**. We assessed study quality (design and implementation) separately for epidemiological, *in vivo*, and *in vitro* studies. Each predefined criterion received one point; the detailed scheme is shown in ***Supplementary Table 4***.

**Correlation score**. We captured the direction of the association between exposure and outcome: positive correlation = 1; no significant correlation = 0; negative correlation = −1.

**Outcome fitness score**. This reflects how strongly the study results support the hypothesized association, categorized as weak (1 point) or strong (2 points). Details are provided in ***Supplementary Table 5***.

**Integrity score**. This reflects the completeness of the overall evidence base. Evidence sets including only one type of study were weighted by 1/3; those including two types were weighted by 2/3; and those including all three types (epidemiological, *in vivo*, and *in vitro*) retained the original score (×1).

The comprehensive evidence score was calculated as follows:

Comprehensive evidence score = [Σ(Reliability score × Correlation score × Outcome fitness score) × Integrity score]/Evidence number.

#### Risk matrix classification

Based on the comprehensive evidence score, chemicals were categorized into three health risk levels using a predefined risk matrix (***Supplementary Table 6***). Scores of (0, 4], (4, 8], and (8, 10] indicate low, moderate, and high risk, respectively. Negative scores reflect protective effects, with [−4, 0), [−8, −4), and [−10, −8) indicating low, moderate, and high levels of protection, respectively.

### Statistical analysis

All statistical analyses were performed using R (version 4.5.1) or GraphPad Prism (version 9.4.1; GraphPad Software, San Diego, CA, USA). Data are presented as mean ± standard deviation unless otherwise specified. Comparisons between two groups were conducted using a two-tailed Student's *t*-test, while multiple-group comparisons were performed using one-way ANOVA followed by Tukey's HSD test; beating proportions were compared using a two-sided Fisher's exact test. In the GO analysis, upregulated and downregulated DEGs were subjected to separate enrichment analyses. A *P* value < 0.05 was considered statistically significant.

## Results

### BPA exposure impaired cardiac organoid development and contractile function

**Figure 1:**
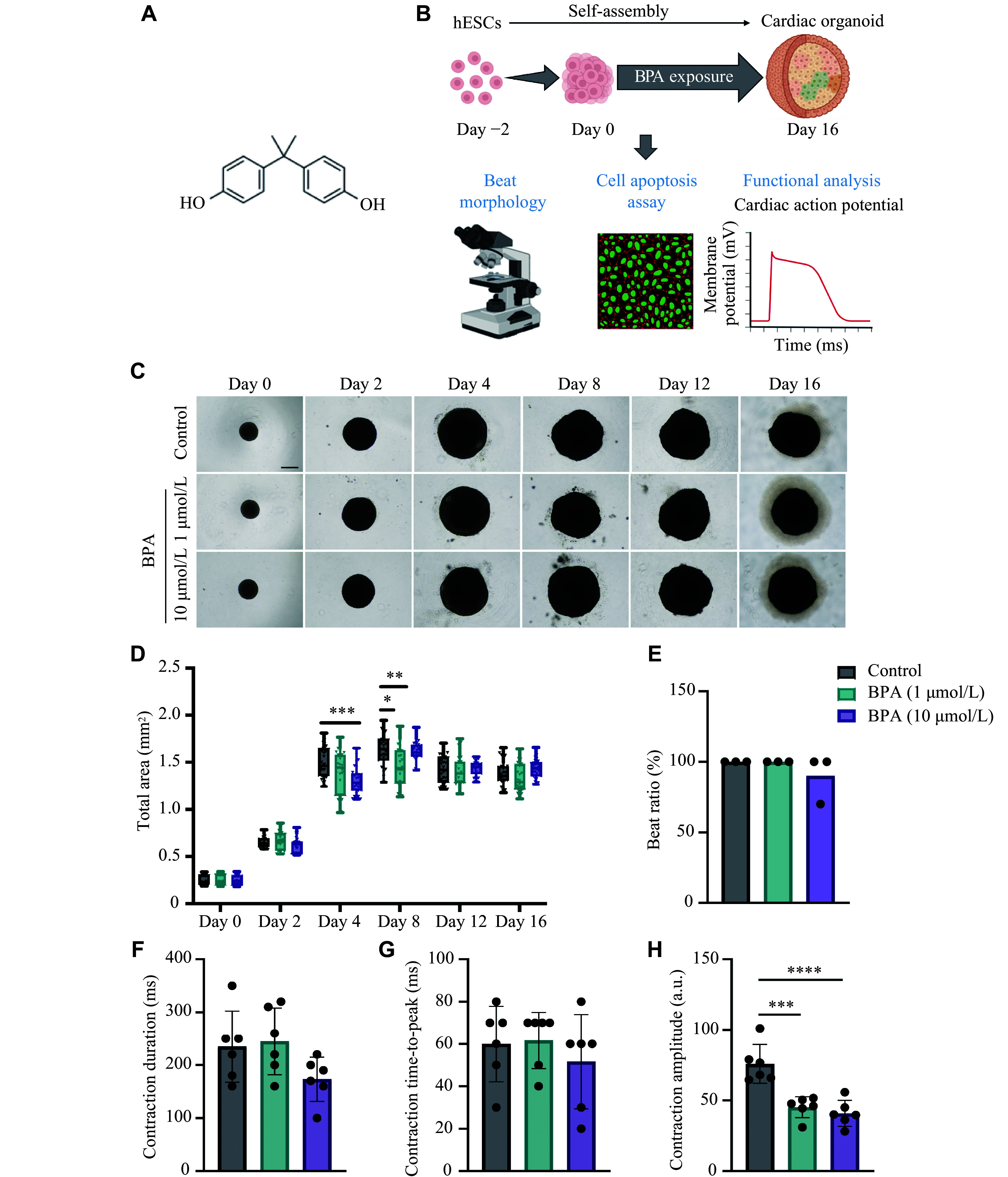
Bisphenol A (BPA) exposure impaired human cardiac organoid (hCO) development and contractile function. A: Chemical structure of BPA (C_15_H_16_O_2_). B: Schematic of BPA exposure and analytical workflow. C: Bright-field images of hCOs at different BPA concentrations and indicated time points. Scale bar: 500 μm. D: Quantification of hCO size. Data are presented as mean ± standard deviation (SD) with individual data points. *n* = 27 hCOs per group derived from three independent differentiation batches. Statistical significance was determined by one-way ANOVA followed by Tukey's HSD test. E: hCO beating activity. Beating was scored as a binary outcome (beating *vs.* non-beating). Data were collected from three independent differentiation batches (*n* = 3), with 10 hCOs per batch per group (30 hCOs per group in total). Group differences in beating proportions were analyzed using a two-sided Fisher's exact test. F–H: Effects of BPA on cardiac contractility, including amplitude, duration, and time-to-peak (*n* = 6 individual hCOs per group from three independent differentiation batches). Data are presented as mean ± SD and analyzed by one-way ANOVA. Post hoc analysis was performed using Tukey's HSD test. ^*^P < 0.05, ^**^P < 0.01, ^***^P < 0.001, and ^****^P < 0.0001.

Based on reported human internal exposure levels, we employed a BPA concentration range of 1.0–10 μmol/L to model a broad range of exposure scenarios, covering low-dose environmental levels to high-dose toxicity thresholds. While urinary concentrations cannot be directly equated to free BPA in cardiac tissue due to differences in metabolism and distribution, neonatal data indicate the potential for significant internal exposure. For instance, Lee *et al*^[6]^ reported maximum BPA concentrations in neonatal urine as high as 946 μg/L (approximately 4.14 μmol/L), and occupational studies have detected urinary levels up to 8.5 μmol/L^[^^22^^]^ in workers with long-term exposure. Furthermore, previous *in vitro* studies have shown that 10 μmol/L BPA significantly reduces the viability of human cardiomyocytes (AC16 cells) after 48 hours. Therefore, we selected lower experimental concentrations (0.1–1 μmol/L), informed in part by reported human biomonitoring data, together with a high-dose condition (10 μmol/L) to capture the range of potential developmental cardiotoxic effects during continuous exposure throughout differentiation (days 0–16; ***Fig. 1A*** and ***1B***).

To establish a robust 3D hCO model for simulating human heart development *in vitro*, we adopted a published differentiation protocol^[16]^. Self-assembling hCOs with cavity-like structures and spontaneous contractions were successfully generated by day 16 (***Supplementary Fig. 1A***). Assessment of gross morphology revealed no obvious differences between BPA-treated and control hCOs throughout the differentiation process (***Fig. 1C***). At the cardiac progenitor stage (day 4), hCOs treated with 10 μmol/L BPA were significantly smaller, a difference that was no longer evident by day 12, suggesting a transient delay in early differentiation that was compensated for during subsequent maturation (***Fig. 1D***).

Functionally, all hCOs in the control and 1 μmol/L BPA groups contracted spontaneously (*n* = 30 per group), whereas 3 of 30 hCOs (10%) in the 10 μmol/L BPA group did not beat, a difference that did not reach statistical significance (***Fig. 1E***). Quantification of contractility using the video-based analysis tool MUSCLEMOTION revealed that 1 μmol/L BPA significantly reduced contraction amplitude without altering contraction duration. In contrast, 10 μmol/L BPA further decreased contraction amplitude, reduced contraction frequency, and induced arrhythmic beating patterns (***Supplementary Fig. 2***). Statistical comparisons demonstrated significant reductions in contraction amplitude in both BPA groups, while time to peak and contraction duration remained unchanged (***Fig. 1F***–***1H***). In summary, these results indicate that 1 μmol/L BPA primarily dampens contraction amplitude, whereas 10 μmol/L BPA induces more severe functional deficits, including decreased frequency and arrhythmia, indicating a pronounced impairment of hCO contractility.

### BPA exposure disrupted endothelial organization and induced apoptosis

**Figure 2:**
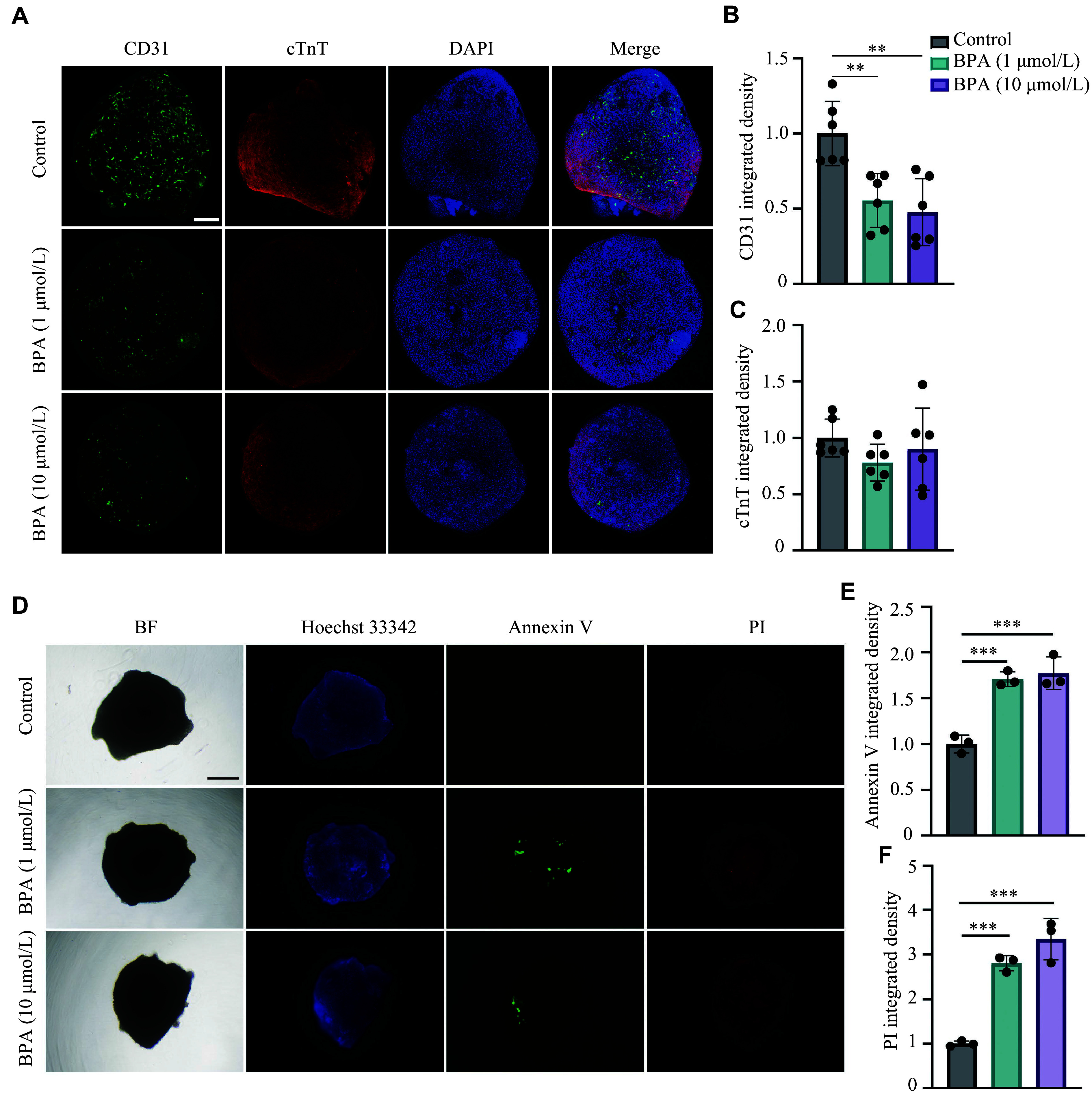
Bisphenol A (BPA) exposure disrupted endothelial organization and increased cell death in human cardiac organoids (hCOs). A: Immunofluorescence staining of cluster of differentiation 31-positive (CD31^+^) endothelial cells (green) and cardiac troponin T-positive (cTnT^+^) cardiomyocytes (red) in hCOs under different BPA concentrations. Scale bar: 200 μm. B and C: Quantification of relative mean fluorescence intensity for CD31 (B) and cTnT (C) (*n* = 6 hCOs per group, derived from three independent differentiation batches; analysis performed at the individual hCO level). Data are presented as mean ± standard deviation (SD) with individual data points. Group differences were analyzed by one-way ANOVA followed by Tukey's HSD test. D: Apoptosis was assessed by Annexin Ⅴ-FITC (green, early apoptosis) and PI (red, late apoptosis/necrosis) staining. Scale bar: 500 μm. E and F: Quantitative analysis of Annexin Ⅴ (E) and PI (F) fluorescence intensity (*n* = 3 hCOs per group, derived from one differentiation batch; analysis performed at the individual hCO level). Data are presented as mean ± SD with individual data points. Group differences were analyzed by one-way ANOVA followed by Tukey's HSD test. ^**^P < 0.01 and ^***^P < 0.001.

To assess the effects of BPA on key cellular components, we performed immunofluorescence staining for the cardiomyocyte marker cardiac troponin T (cTnT) and the endothelial marker cluster of differentiation 31 (CD31) in day-16 hCOs. We observed no obvious difference in cTnT staining between BPA-treated groups and controls, indicating that cardiomyocyte differentiation was not impaired. In contrast, both 1 μmol/L and 10 μmol/L BPA exposure markedly weakened CD31 fluorescence signals and disrupted endothelial network integrity, as evidenced by a loss of interconnected vascular-like structures. Quantitative analysis of fluorescence intensity supported these observations (***Fig. 2A***–***2C***). However, mRNA expression of cardiac troponin T type 2 (*TNNT2*), platelet and endothelial cell adhesion molecule 1 (*PECAM1*/*CD31*), and vimentin (*VIM*) remained unchanged across groups (***Supplementary Fig. 3***), suggesting that the observed reduction in CD31 staining and network organization was not accompanied by corresponding changes in the transcript levels of the examined lineage markers. Together, these data show that BPA exposure does not impair cardiomyocyte specification but significantly affects CD31^+^ ECs in hCOs.

We also examined apoptosis by Annexin Ⅴ-FITC/PI staining. Treatment with 1 μmol/L and 10 μmol/L BPA resulted in enhanced Annexin Ⅴ and PI fluorescence signals, indicating Annexin Ⅴ positivity and loss of membrane integrity, consistent with increased apoptotic and/or necrotic cell death (***Fig. 2D***). Quantitative data showed that BPA significantly increased both Annexin Ⅴ and PI signals compared with controls (***Fig. 2E*** and ***2F***). These results demonstrate that BPA exposure increases cell death-associated signals, consistent with enhanced apoptosis and/or necrosis, in hCOs. Notably, the concentrations at which BPA induced apoptosis were consistent with those causing endothelial impairment, suggesting a potential role for cell death in BPA-mediated vascular injury.

### Transcriptional alterations underlying BPA-induced cardiotoxicity

**Figure 3:**
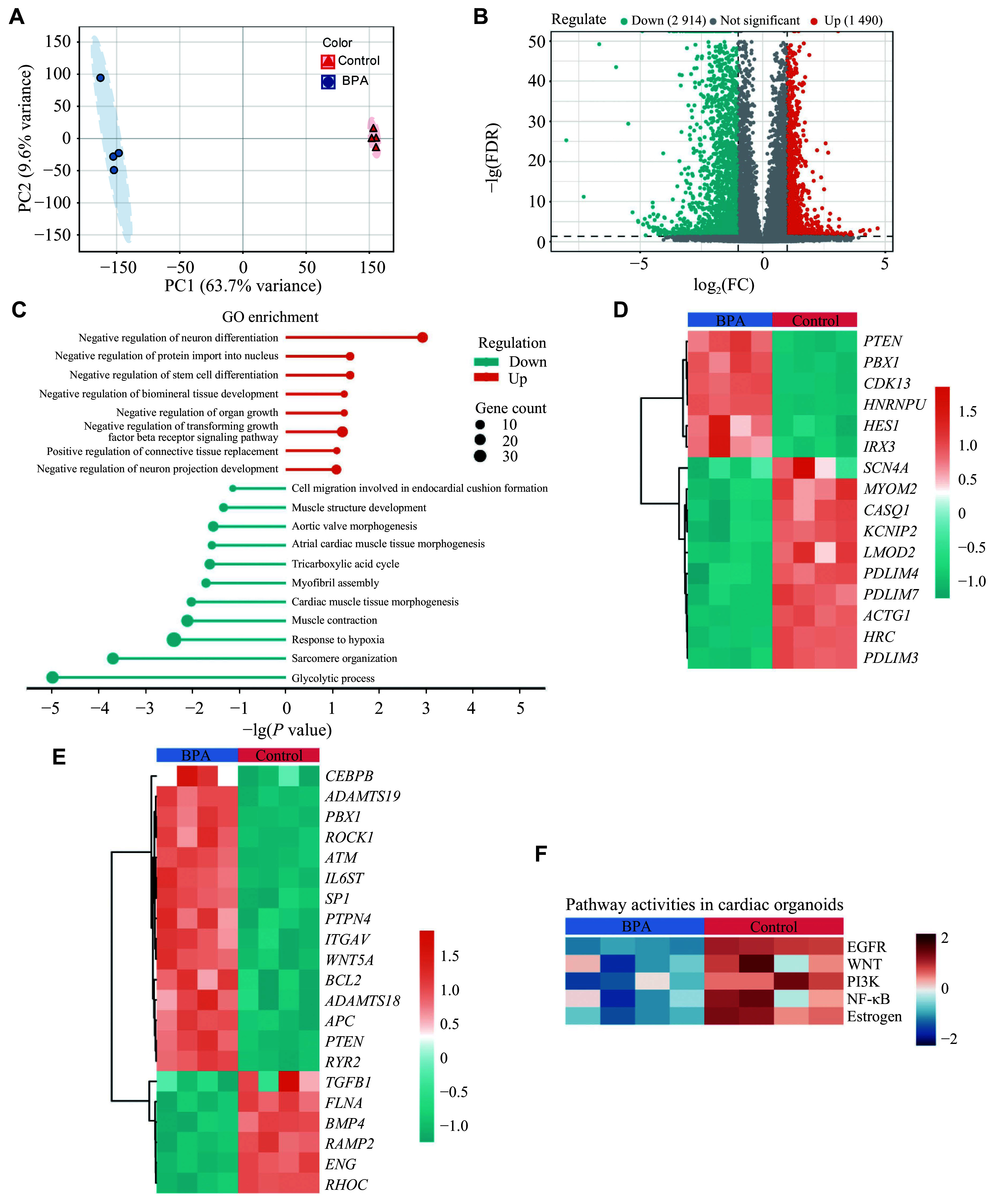
Transcriptional alterations associated with bisphenol A (BPA)-induced cardiotoxicity. A: Principal component analysis of RNA-seq data from control and 1 μmol/L BPA-treated human cardiac organoids (four independent biological replicates). PC1 and PC2 denote the first and second principal components, respectively. B: Volcano plot of differentially expressed genes (DEGs) (false discovery rate [FDR] < 0.05, |log_2_FC| > 1), with 1490 genes upregulated (red) and 2914 genes downregulated (blue). C: Gene Ontology (GO) enrichment analysis of DEGs, focusing on biological processes and molecular functions related to cardiac differentiation and function. D: Heatmap of DEGs clustered by expression pattern across samples. E: Heatmap showing the expression profiles of selected genes in cardiac organoids. F: Heatmap showing normalized PROGENy pathway activity scores inferred from bulk RNA-seq data of day-16 cardiac organoids treated with 1 μmol/L BPA or control.

To explore the underlying mechanistic changes, we performed RNA-seq on hCOs exposed to 1 μmol/L BPA for 16 days. PCA revealed clear separation and distinct clustering between control and BPA-treated hCOs (***Fig. 3A***). BPA exposure altered the expression of 4404 genes, with 1490 upregulated and 2914 downregulated (***Fig. 3B***). GO enrichment analysis indicated that BPA affected biological processes related to cardiac rhythm, organogenesis, and stem cell differentiation (***Fig. 3C***). Dysregulation of key rhythm-associated genes (*e.g.*, upregulation of *HES1*, *IRX3*, and *PBX1*; downregulation of *MYOM2*, *ACTG1*, and *LMOD2*; ***Fig. 3D***) was consistent with the observed contractile dysfunction.

Unsupervised hierarchical clustering of selected genes also revealed a clear separation between control and BPA-treated hCOs. Examination of these genes demonstrated increased expression of ECM-related metalloproteinases such as *ADAMTS18* and *ADAMTS19*, as well as inflammatory regulators including *CEBPB* and *IL6ST*. Expression levels of genes linked to cellular stress and survival, such as *ATM* and *PTEN*, and genes implicated in cardiac function and development, including *RYR2*, *WNT5A*, and *APC*, were also altered. These trends may reflect changes in ECM organization, inflammatory signaling, and cardiac regulatory pathways in response to BPA, although additional functional studies are needed to clarify their specific roles in BPA-induced injury (***Fig. 3E***).

To gain an overview of upstream signaling changes, we applied the Projection of Gene Network y (PROGENy)-based pathway analysis and observed the reduced inferred activity of the epidermal growth factor receptor (EGFR), WNT, and phosphatidylinositol 3-kinase (PI3K) pathways, together with altered estrogen-related pathway activity in BPA-treated hCOs (***Fig. 3F***). Collectively, these findings indicate that BPA exposure might influence multiple signaling pathways involved in cardiac development.

GSEA further indicated the downregulation of several cardiac development-related gene sets, including "regulation of mesodermal cell differentiation", "cardiac muscle cell fate commitment", and "atrial cardiac muscle tissue development" (***Supplementary Fig. 4A***–***4C***). Additionally, the "tricarboxylic acid cycle" gene set showed negative enrichment, suggesting an alteration of energy metabolism (***Supplementary Fig. 4D***), which may contribute to the contractile abnormalities observed in BPA-treated hCOs.

### BPA disrupted vascular network formation in hCOs

**Figure 4:**
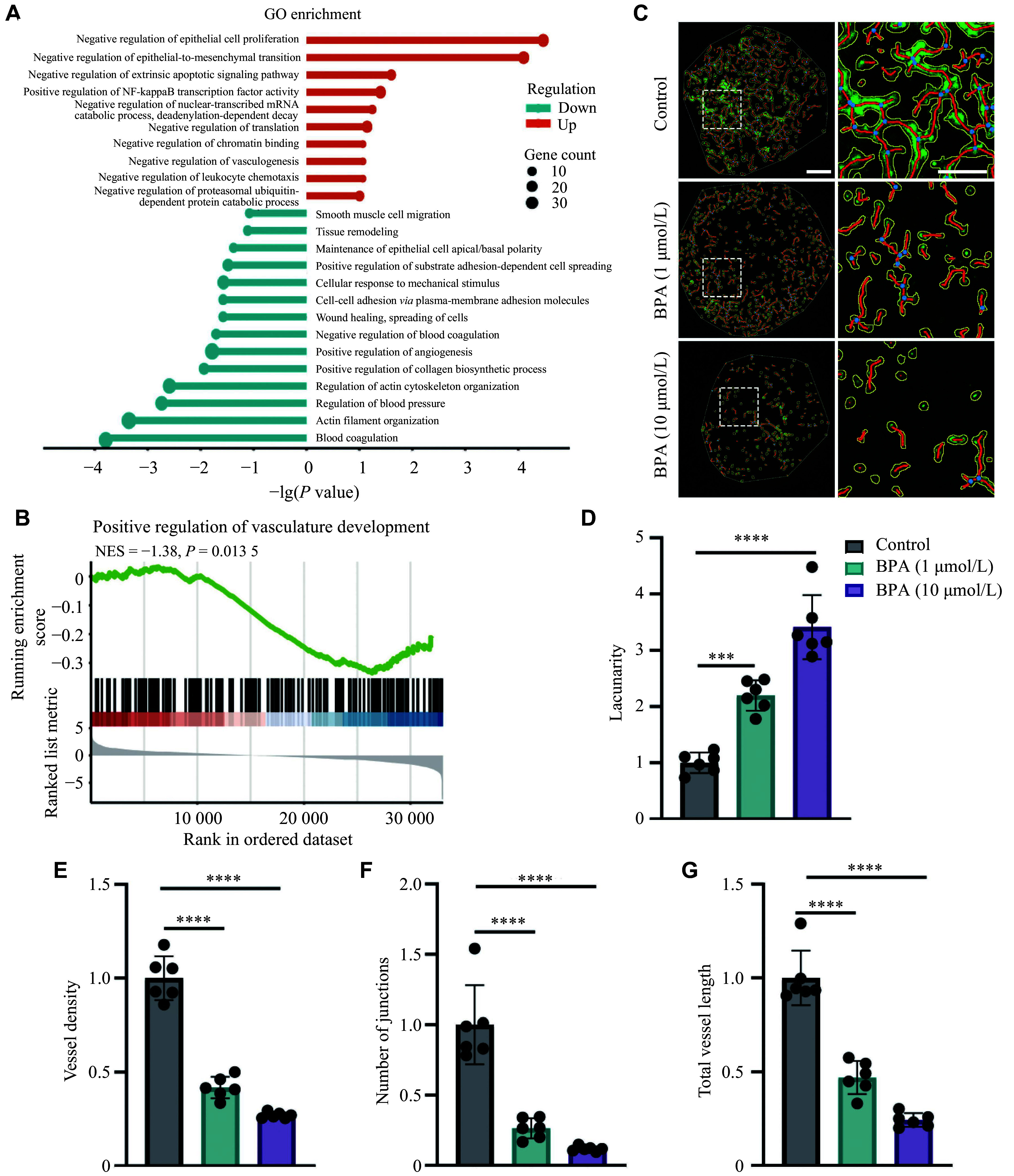
BPA disrupted vascular network formation in human cardiac organoids (hCOs). A: Gene Ontology (GO) enrichment analysis of differentially expressed genes (DEGs), showing the significantly enriched biological processes and molecular functions. B: Gene set enrichment analysis plots showing significant suppression of regulation of vasculature development (normalized enrichment score [NES] = −1.38, *P* = 0.0135). C: Representative CD31 immunofluorescence images of hCOs from different BPA treatment groups, analyzed by AngioTool. Left panels show original images with vessel contours (yellow), skeletons (red), and branch points (blue). Right panels display magnified views of the dashed-boxed regions in the corresponding left panels. Scale bars: 200 μm (left) and 100 μm (right). D–G: Quantitative analysis of vascular parameters: lacunarity (D), vessel density (E) (vessel area/hCO area), number of vessel junctions (F), and total vessel length (G). *n* = 6; *n* indicates the number of individual hCOs analyzed. Data are presented as mean ± standard deviation and analyzed by one-way analysis of variance. Post hoc analysis was performed using Tukey's HSD test. ^***^P < 0.001 and ^****^P < 0.0001.

Consistent with the observed loss of CD31^+^ ECs, transcriptomic GO analysis showed the downregulation of "positive regulation of angiogenesis" and the upregulation of "negative regulation of vasculogenesis" (***Fig. 4A***). BPA exposure also altered the expression of key genes involved in endothelial differentiation and vascular patterning, including the upregulation of *PTEN* and the downregulation of *ENG* and *BMP4* (***Fig. 3E***). GSEA further supported these findings, showing significant suppression of gene sets related to vasculature development (***Fig. 4B***). These results collectively indicate that BPA exposure perturbs transcriptional programs essential for vessel development and vascular network formation.

To further evaluate the impact of BPA on vascular network structure, we performed a quantitative analysis of hCOs using AngioTool software^[23]^. Control hCOs exhibited well-connected and continuous endothelial networks with abundant branching junctions. In contrast, both 1 μmol/L and 10 μmol/L BPA-treated hCOs showed fragmented networks characterized by reduced vessel density, increased intervascular spaces, and diminished branching complexity (***Fig. 4C***). Quantification confirmed that BPA exposure significantly increased lacunarity, decreased vascular density, reduced junction points, and shortened total vessel length compared with controls (***Fig. 4D***–***4G***). These findings demonstrate that BPA exposure impairs vascular network formation in hCOs by reducing vessel complexity, connectivity, and overall integrity, thereby compromising hCO differentiation and maturation.

### Identification of a molecular sensitivity point for BPA-induced endothelial impairment in hCOs

**Figure 5:**
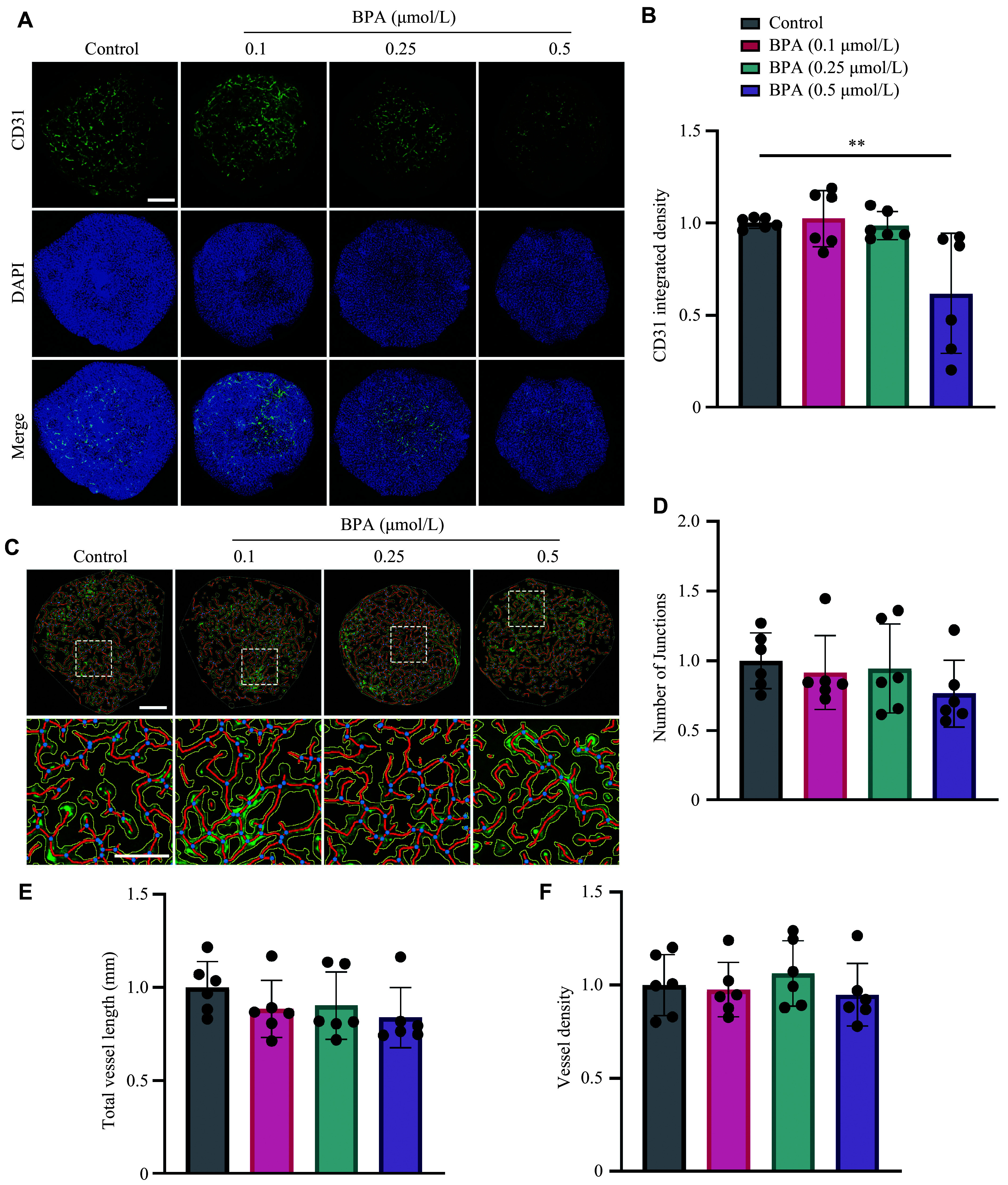
Dose-dependent effects of bisphenol A (BPA) exposure on endothelial networks in human cardiac organoids (hCOs). A: Representative immunofluorescence images showing cluster of differentiation 31-positive (CD31^+^) endothelial cells (green) in hCOs following exposure to decreasing concentrations of BPA. Scale bar: 200 μm. B: Quantitative analysis of the relative mean fluorescence intensity of CD31 staining (*n* = 6). C: AngioTool-processed images of CD31-stained hCOs. Top: Full views showing vessel outlines (yellow), skeletons (red), and branch points (blue). Bottom: Magnifications of the dashed-boxed areas. Scale bars: 200 μm (top) and 100 μm (bottom). D–F: Quantitative assessment of vessel junctions (D), total vessel length (E), and vessel density (F) (vessel area/hCO area) across BPA concentrations (*n* = 6). In B and D–F, *n* indicates the number of individual hCOs analyzed. Data are presented as mean ± standard deviation and analyzed by one-way analysis of variance. Post hoc analysis was performed using Tukey's HSD test. ^**^P < 0.01.

Based on the observed suppression of CD31^+^ ECs and disruption of vascular networks at 1 μmol/L and 10 μmol/L BPA, we further investigated whether lower concentrations would elicit similar effects. hCOs were exposed to 0.1, 0.25, or 0.5 μmol/L BPA throughout differentiation. Immunofluorescence staining revealed that CD31^+^ ECs remained comparable to controls and formed well-connected networks at 0.1 and 0.25 μmol/L BPA, comparable with controls. In contrast, the 0.5 μmol/L BPA group showed a marked reduction in CD31 fluorescence, along with disruptions in network continuity (***Fig. 5A***). Quantitative analysis confirmed a significant decrease in CD31 intensity at 0.5 μmol/L BPA (***Fig. 5B***). AngioTool-based quantification indicated that vascular networks in the 0.1 and 0.25 μmol/L groups retained normal complexity, with no significant differences in vascular density, junction number, or total vessel length compared with controls. Although the 0.5 μmol/L group exhibited a slight reduction in vascular junctions, the difference was not statistically significant, and other parameters remained unchanged (***Fig. 5C***–***5F***). Among the concentrations tested, 0.5 μmol/L was the lowest concentration associated with a significant reduction in CD31 fluorescence intensity, although other quantitative vascular-network parameters were not significantly altered.

### TRAEC-based risk assessment and cross-chemical screening highlighted BPA and related EDCs as potential cardiotoxins

**Figure 6:**
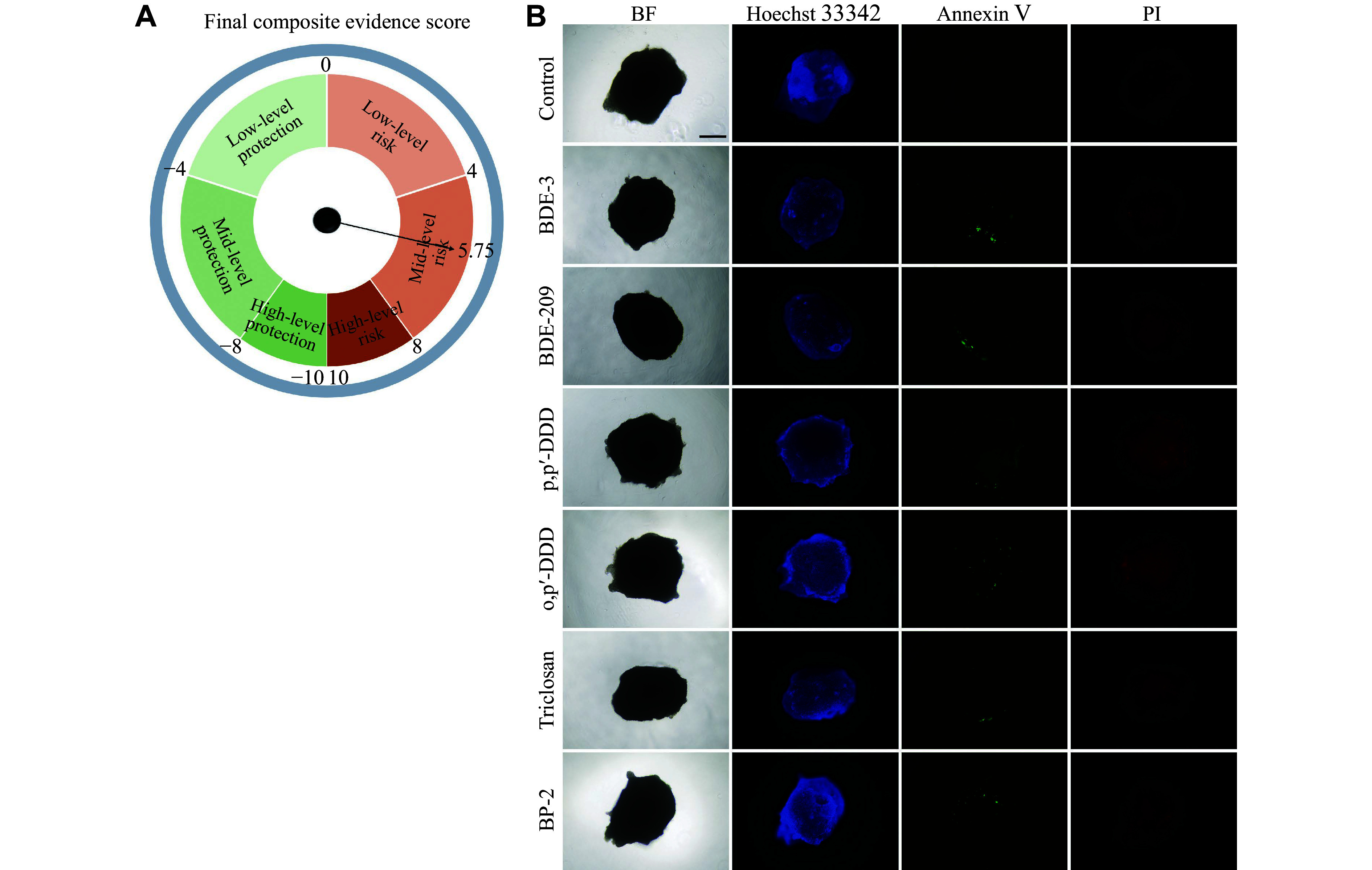
TRAEC-based risk assessment and cross-chemical screening highlighted bisphenol A and related EDCs as potential cardiotoxins. A: The circular diagram illustrates the integrated evidence score derived from all assessed studies (***Supplementary Tables 7***–***9*** and original data). The radial axis represents the score values ranging from −10 to 10. The sectors are color-coded to indicate the degree of protection (green, left hemisphere) or risk (orange-red, right hemisphere), further categorized into low, mid, and high levels. The black arrow points to a final composite score of 5.75, indicating an overall mid-level risk of cardiotoxicity for BPA. All evaluations were performed according to TRAEC guideline specifications. B: Representative images of Annexin Ⅴ-FITC and PI staining in human cardiac organoids treated with different EDCs. Scale bar: 500 μm. Abbreviations: EDCs, endocrine-disrupting chemicals; TRAEC, Targeted Risk Assessment of Environmental Chemicals.

To systematically evaluate the cardiac developmental risk associated with BPA, we employed the TRAEC framework. The literature search identified 12 relevant studies (***Supplementary Fig. 5A***; ***Supplementary Tables 7***–***9***)^[7-10,24-31]^. Because TRAEC scores evidence units rather than studies, one publication^[^^8^^]^ reporting both *in vivo* and *in vitro* data was scored as two units. Thus, 12 published studies contributed 13 evidence units, and together with one unit from our study, 14 evidence units were included. Each unit was scored for reliability, correlation, and outcome fitness (***Supplementary Fig. 5B***–***5D***; criteria in ***Supplementary Tables 4***–***6***) by two independent reviewers with third-party adjudication. Reliability scores ranged from 3 to 5 points (***Supplementary Fig. 5B***), while all studies demonstrated a consistent positive association between BPA exposure and adverse cardiac developmental outcomes, yielding a correlation score of 1 point (***Supplementary Fig. 5C***). Outcome fitness scores varied from 1 to 2 points (***Supplementary Fig. 5D***). Based on these criteria, individual evidence scores were calculated, producing values of 10, 4.5, 8, 4.5, 4, 4, 6.5, 8, 4, 3, 3.5, 6, 4.5, and 10 (***Supplementary Fig. 5E***). Integrity was 100% because evidence covered epidemiological, *in vivo*, and *in vitro* streams (***Supplementary Tables 7***–***9***). The comprehensive evidence score was calculated as [Σ (reliability × correlation × outcome fitness) × integrity]/evidence number. With an evidence number of 14, the overall score was 5.75, indicating a moderate level of cardiac developmental risk associated with BPA exposure (***Fig. 6A***).

Guided by this risk indication, we further investigated whether structurally related EDCs, including 4-bromodiphenyl ether (BDE-3), 2,2′,3,3′,4,4′,5,5′,6,6′-Decabromodiphenyl ether (BDE-209), dichlorodiphenyldichloroethane (p,p$ {'} $-DDD), 2,4$ {'} $-dichlorodiphenyldichloroethane (o,p${'} $-DDD), triclosan, and benzophenone-2 (BP-2), may elicit similar cardiotoxicity. These compounds have been detected in maternal amniotic fluid or umbilical cord blood (***Supplementary Fig. 6A***–***6F***)^[^^32^^–^^33^^]^. hCOs exposed to each EDC at ten times their reported human detection levels (days 0–16) showed no significant differences in organoid size, morphology, or spontaneous contraction (***Supplementary Fig. 6G***). However, Annexin Ⅴ-FITC/PI staining revealed mild increases in early apoptosis in hCOs treated with BDE-3, BDE-209, o,p′-DDD, triclosan, or BP-2. Late apoptosis/necrosis was elevated in groups exposed to p,p′-DDD, o,p′-DDD, or BP-2 (***Fig. 6B***). Notably, o,p′-DDD and BP-2 induced apoptotic phenomena similar to those triggered by BPA, suggesting that these EDCs may warrant further investigation for potential developmental cardiotoxicity. Because the current hCO study was included as one evidence unit in the TRAEC calculation, the integrated score should not be considered fully independent validation of the experimental findings.

## Discussion

Organoids and organ-on-a-chip systems have emerged as powerful platforms for toxicological evaluation, providing human-relevant insights into how environmental exposures perturb early organ development^[^^34^^]^. Using a human ESC-derived hCO model, we investigated whether BPA impaired cardiac development and observed dose-dependent disruptions in contractile function and endothelial organization. These functional deficits align with accumulating evidence linking BPA exposure to congenital heart defects.

BPA exposure reduced contraction amplitude and, at higher doses, induced rhythm instability, suggesting early impairment of the excitation-contraction machinery. Transcriptomic analysis revealed altered expression of genes associated with cardiac stress and remodeling, including the upregulation of ECM-related metalloproteinases (*e.g.*, *ADAMTS18* and *ADAMTS19*) and inflammatory regulators (*IL6ST*), indicating the initiation of a remodeling-like response during early development. Dysregulation of survival pathways (*e.g.*, *ROCK1* and *BCL2*) further supports a shift toward a stressed, apoptosis-prone state. Upregulation of *ATM* and *PTEN* reinforces this pro-apoptotic profile, consistent with the observed increase in apoptosis. Moreover, BPA altered the expression of genes central to cardiac electrophysiology and development, such as *RYR2* and components of WNT signaling (*e.g.*, *WNT5A* and *APC*), linking transcriptional disturbance to the contractile deficits observed in hCOs. These findings are consistent with previous reports of BPA-induced calcium dysregulation and contractile impairment in hPSC-CMs^[^^11^^,^^13^^]^.

Beyond contractile defects, endothelial damage emerged as a key phenotype. BPA significantly impaired CD31^+^ ECs and disrupted vascular network formation, mirroring *in vivo* evidence that prenatal BPA exposure impairs cardiac vasculogenesis and vascular remodeling. As transcriptomic changes did not indicate substantial suppression of endothelial lineage gene expression, these vascular defects may result from post-transcriptional mechanisms. Previous studies have also reported that gestational low-dose exposure to bisphenol S enhances nitric oxide (NO)-mediated vasodilatory responses in female offspring mice, suggesting that BPA and its structural analogs may induce broader endothelial dysfunction beyond apoptosis, leading to systemic vascular dysregulation^[^^35^^]^. Given that apoptosis was assessed at the whole-organoid level, future lineage-specific analyses (*e.g.*, Annexin Ⅴ/CD31 co-staining) are needed to determine whether ECs are disproportionately vulnerable.

As a selective estrogen receptor modulator, BPA is known to interact with estrogen receptors (ERs), including ERα, ERβ, and G protein-coupled estrogen receptor (GPER). Imbalanced ER signaling during early development has been linked to altered inflammatory tone, disrupted redox homeostasis, and cardiac malformations, including pericardial edema^[^^36^^]^. Numerous studies have indicated that ER dysregulation underlies the sex-dependent cardiovascular effects of BPA. In fetal hearts, BPA decreases ERα in females but increases it in males^[^^37^^]^, which may respectively disturb normal cardiac programming or sensitize hearts to adverse responses under stress^[^^38^^]^. ERβ plays a vital role in maintaining cardiac homeostasis by mitigating inflammation and modulating oxidative stress^[^^39^^]^. Prenatal BPA exposure in mice reduces ERβ expression, thereby promoting a pro-inflammatory and pro-oxidative environment during cardiac development^[^^37^^]^. Consistent with these data, our PROGENy analysis indicated altered estrogen-related pathway activity, supporting further investigation of receptor-specific mechanisms involving ERα, ERβ, and GPER. These findings highlight the need for receptor-specific analyses in human-relevant models and for explicitly incorporating sex as an independent variable in future studies.

To contextualize these findings within a broader risk framework, we applied the TRAEC approach to integrate epidemiological, animal, and *in vitro* evidence. The resulting score indicated a moderate level of developmental cardiac risk associated with BPA exposure. While this quantitative framework provides transparency and systematic evaluation, we acknowledge that the conclusions must be interpreted cautiously given uncertainties in exposure relevance and dataset heterogeneity.

Several limitations should also be acknowledged. First, although the exposure concentrations used were informed by neonatal urinary BPA levels and prior *in vitro* studies, they do not directly correspond to free BPA levels in fetal tissues due to differences in metabolism, protein binding, and bioavailability. Continuous exposure throughout organoid differentiation may also overestimate physiological exposure intensity. Second, while hCOs recapitulate key human developmental features, they lack the complex endocrine, inflammatory, and redox microenvironment of the fetus. *In vivo* studies indicate that maternal–fetal transport processes and sex-dependent hormonal contexts modulate cardiac sensitivity to BPA, but such sex-specific dynamics cannot be captured in our hCOs^[^^36^^]^. Finally, apoptosis was evaluated only at the whole-organoid level. However, some phenotypic analyses were based on multiple organoids derived from the same differentiation batch, and future studies with larger numbers of independent differentiation batches are needed to confirm the robustness of these findings. Future work incorporating lineage-resolved apoptosis markers will be important for clarifying cell-lineage-specific vulnerability.

Collectively, the current study shows that BPA exposure impairs early human cardiac development in human ESC-derived hCOs, affecting contractile performance and endothelial organization alongside broad transcriptional disturbances in cardiac rhythm and vascular developmental pathways. Integrating these findings with existing literature *via* the TRAEC framework indicates a moderate developmental cardiac risk associated with BPA exposure. In exploratory screening, o,p′-DDD and BP-2 produced cell death-associated staining patterns resembling those observed with BPA, warranting further investigation of their potential developmental cardiotoxicity. While hCOs provide a valuable platform for environmental toxicity assessment, limitations in physiological maturity and the translation of *in vitro* doses to *in vivo* exposure conditions underscore the need for complementary *in vivo* validation. Furthermore, future studies to define cell-type-specific vulnerability and refine developmental risk assessment will also be essential. Together, these findings highlight how BPA and related EDCs may influence fetal cardiac development and support the continued refinement of organoid-based toxicity testing systems.

## Additional information

The online version contains supplementary material available at http://www.jbr-pub.org.cn/article/doi/10.7555/JBR.39.20250533.
