# Supplementary material for: Bisphenol A impairs contractile function and vascular development in human cardiac organoids: Insights from toxicity assessment and evidence-based risk evaluation
Source: J Biomed Res. 2026 Jul 28;40(4):448–62. doi: 10.7555/JBR.39.20250533 (PMC13438319; doi:10.7555/JBR.39.20250533)
Supplement: Supplementary file 1 [file jbr-40-4-448-S1.pdf]

Available online at www.jbr-pub.org.cn

Open Access at PubMed Central

Journal of Biomedical Research, 2026 40(4): 1–8

Supplementary Data

JBR

Bisphenol A impairs contractile function and vascular development in human cardiac organoids: Insights from toxicity assessment and evidence-based risk evaluation

Linfeng Jiang[1,△], Yachang Wang[1,△], Huina Li[1], Yang Yang[1,2,✉]

[1]*State Key Laboratory of Reproductive Medicine and Offspring Health, Nanjing Medical University, Nanjing, Jiangsu 211166, China;*
[2]*Jiangsu Environmental Health Risk Assessment Engineering Research Center, Key Laboratory of Modern Toxicology of Ministry of Education, Center for Global Health, Nanjing Medical University, Nanjing, Jiangsu 211166, China.*

Supplementary Table 1 **Detailed information of endocrine-disrupting chemicals (EDCs)**

EDCs	Catalog number	Manufacturer	Source
Bisphenol A (BPA)	P109919	Sinopharm	Shanghai, China
4-Bromodiphenyl ether (BDE-3)	B831387	Macklin	Shanghai, China
2,2′,3,3′,4,4′,5,5′,6,6′-Decabromodiphenyl ether (BDE-209)	D807059	Macklin	Shanghai, China
Dichlorodiphenyldichloroethane (p,p′-DDD)	B807766	Macklin	Shanghai, China
2,4′-Dichlorodiphenyldichloroethane (o,p′-DDD)	M833830	Macklin	Shanghai, China
Triclosan	T107430	Aladdin	Shanghai, China
Benzophenone-2 (BP-2)	T838464	Macklin	Shanghai, China

Supplementary Table 2 **The sequences of primers used for reverse transcription-quantitative PCR**

Primers	Sequences (5′–3′)
hq-*PECAM1*-F1	AACAGTGTTGACATGAAGAGCC
hq-*PECAM1*-R1	TGTAAAACAGCACGTCATCCTT
hq-*TNNT2*-F1	ACAGGAGGAAGGCTGAGGAT
hq-TN*NT2*-R1	CGAACTTCTCTGCCTCCAAG
hq-*VIM*-F1	AGTCCACTGAGTACCGGAGAC
hq-*VIM*-R1	CATTTCACGCATCTGGCGTTC
hq-*GAPDH*-F1	CCCTTTTGGCTCCACCCT
hq-*GAPDH*-R1	TTCACCACCATGGAGAAGGC

The prefix "hq-" indicates human-specific qPCR primers.

△These authors contributed equally to this work.

✉Corresponding author: Yang Yang, E-mail: yangyang11@njmu.edu.cn, ORCID: 0000-0002-9887-7921.

Received: 29 November 2025; Revised: 08 March 2026; Accepted: 12 March 2026; Available online: 30 March 2026; Published date: 28 July 2026

CLC number: R114, Document code: A

The authors reported no conflicts of interest.

This is an open access article under the Creative Commons Attribution (CC BY 4.0) license, which permits others to distribute, remix, adapt and build upon this work, for commercial use, provided the original work is properly cited.

© 2026 by Journal of Biomedical Research.

https://doi.org/10.7555/JBR.39.20250533

Supplementary Table 3 **Antibody information**

Antibodies	Catalog number	Manufacturer	Diluted ratio	Source
Rabbit polyclonal anti-Cardiac Troponin T	ab45932	Abcam	1 ∶ 1 000	Cambridge, UK
Sheep anti-Human CD31/PECAM-1 Antigen Affinity-purified Polyclonal Antibody	AF806	R&D Systems	1 ∶ 1 000	Minneapolis, MN, USA
Vimentin Rabbit mAb	A19607	ABclonal	1 ∶ 1 000	Wuhan, China
Donkey anti-Rabbit IgG (H+L) Highly Cross-Adsorbed Secondary Antibody, Alexa Fluor 555	A31572	Invitrogen	1 ∶ 1 000	Carlsbad, CA, USA
Donkey anti-Mouse IgG (H+L) ReadyProbes Secondary Antibody, Alexa Fluor 555	A31570	Invitrogen	1 ∶ 1 000	Carlsbad, CA, USA
Donkey anti-Goat IgG (H+L) Cross-Adsorbed Secondary Antibody, Alexa Fluor 488	A11055	Invitrogen	1 ∶ 1 000	Carlsbad, CA, USA
Donkey anti-Sheep IgG (H+L) Cross-Adsorbed Secondary Antibody, Alexa Fluor 488	A-11015	Invitrogen	1 ∶ 1 000	Carlsbad, CA, USA

Supplementary Table 4 **Reliability scoring criteria**

Study type	Scoring criteria
Epidemiological study	The study was designed appropriately and effectively to achieve the research objectives.
	Exposure details or information were collected, including population baseline data, exposure levels, and confounding factors.
	Reliable and valid tests and/or measurement methods were defined to determine endpoints or outcomes.
	Data sources and/or results (hazard ratio [HR], relative risk [RR], or odds ratio [OR]) for effect estimation were collected and recorded, such as detection data, monitoring datasets, and measurement/supervision of exposure.
	Appropriate and reasonable statistical analyses were performed for measurement, confounding, and sensitivity analysis.
In vivo study	The test compound was specified or identified in the study.
	Reliable and sensitive experimental animals were selected, meeting the requirements of the experimental purpose.
	The exposure to the compound was designed reasonably, with a clear specification of exposure duration, dose, and route, *etc*.
	Reliable and valid tests and/or methods were employed to determine experimental endpoints or outcomes.
	Appropriate and reasonable statistical analyses were performed for measurements and data.
In vitro study	The test compound was specified or identified in the study.
	A reliable and sensitive detection system (*e.g.*, cell line/cell/organoid/subcellular component) was selected to meet the requirements of the experimental purpose.
	The exposure to the compound was designed reasonably, with a clear specification of exposure duration, dose, and route, *etc*.
	Reliable and valid tests and/or methods were employed to determine experimental endpoints or outcomes.
	Appropriate and accurate statistical analyses were performed for measurements and data.

Supplementary Table 5 **Outcome fitness scores**

Study type	Scoring criteria	
Epidemiological study	1	Weak association exists between exposure and outcome (*e.g.*, low odds ratio, relative risk, hazard ratio, *etc.*).
	2	Strong association exists between exposure and outcome (*e.g.*, high odds ratio, relative risk, hazard ratio, *etc.*).
In vivo study	1	Only molecular changes.
	2	Phenotypic and molecular changes.
In vitro study	1	Only molecular changes.
	2	Phenotypic and molecular changes.

Supplementary Table 6 **Risk matrix division**

Reliability scores	Correlation fit scores: the direction of the association between exposure and outcome			
	Very likely negative correlation (−2)	Possible negative correlation (−1)	Possible positive correlation (1)	Very likely positive correlation (2)
Extremely high (4, 5]	High-protection [−10, −8)	Mid-protection [−5, −4)	Mid-risk (4, 5]	High-risk (8, 10]
High (3, 4]	Mid-protection [−8, −6)	Low-protection [−4, −3)	Low-risk (3, 4]	Mid-risk (6, 8]
Medium (2, 3]	Mid-protection [−6, −4)	Low-protection [−3, −2)	Low-risk (2, 3]	Mid-risk (4, 6]
Low (1, 2]	Low-protection [−4, −2)	Low-protection [−2, −1)	Low-risk (1, 2]	Low-risk (2, 4]
Extremely low (0, 1]	Low-protection [−2, 0)	Low-protection [−1, 0)	Low-risk (0, 1]	Low-risk (0, 2]

Supplementary Table 7 **The effects of bisphenol A (BPA) exposure on cardiac development: An epidemiological perspective**

Number	Experimental subject	Quantity and type	Research results	Reference
1	Data from the Portuguese population-based birth cohort Generation XXI was used in the present study.	A convenience subsample of adolescents (*n* = 240) was invited to collect a 24-h urine sample, as close as possible to the interview, 30 days after the interview.	Early BPA exposure can impair cardiometabolic outcomes in the future, namely higher blood insulin levels, insulin resistance, fat mass percentage, waist circumference, and z-score of body mass index.	Costa *et al* (2024)[24]
2	This study investigated 132 children and adolescents aged 6–18 years living in Isfahan, Iran.	Total BPA was detected in urine samples of all participants.	A significant association between BPA exposure and obesity and some cardiometabolic risk factors in children and adolescents.	Amin *et al* (2019)[25]

Supplementary Table 8 **The cardiac developmental toxicity of bisphenol A (BPA) assessed in *in vivo* models**

Number	Experimental subject	Exposure mode	Results	Reference
1	C57BL/6J mice	Mice were exposed to low-dose BPA [40 mg/(kg body weight)] and high-dose BPA [(120 mg/(kg body weight)] for 18 days during pregnancy.	Structural abnormalities in the fetal heart were evident in BPA-exposed mice. These alterations were associated with upregulation of NK2 homeobox 5 and the activation of ferroptosis *in vivo*.	Chen *et al* (2023)[7]
2	Sprague-Dawley rats	In this study, pregnant rats were divided into three experimental groups: vehicle control (0.4% Tween-80), 5 ppm BPA, and 20 ppm BPA, administered in drinking water. The exposure commenced on pregnancy day 2 and continued until the end of gestation, on pregnancy day 21.	Cardiac histology indicated muscle injury in BPA-treated pregnant rats. Furthermore, examination of fetal hearts showed fibrotic signs relative to controls. These structural alterations may be attributable to dysregulated miRNA expression, which adversely impacts cardiac tissue development.	Rasdi *et al* (2020)[26]
3	Sprague-Dawley rats	Pregnant rats were exposed from gestational day 0 for 19 days to either BPA (2 or 100 μg/L), PFOS (40 or 2 000 μg/L), or a combination of both chemicals at corresponding low and high doses.	The co-exposure to PFOS and BPA was observed to induce thickening of the interventricular septum and elevated collagen deposition in fetal rat hearts.	Zhou *et al* (2020)[8]
4	Zebrafish (*Danio rerio*)	Following fertilization, embryos were continuously exposed to either the vehicle, 2 000 μg/L BPA, or 4 000 μg/L BPA for a duration of 24 h.	Exposure to BPA during early embryo life impairs cardiogenesis. Treatment with epigallocatechin gallate restores normal cardiogenesis.	Lombó *et al* (2019)[27]
5	Zebrafish (*Danio rerio*)	Zebrafish embryos/larvae were exposed in the laboratory from 6 h post fertilization (hpf) to 5 days post fertilization (dpf) or from 6 hpf to 15 dpf, to aqueous concentrations of BPA (low 100; high 1 000 μg/L) or its metabolite MBP (low 2.5; high 25 μg/L).	Heart valves were primary sites for ERE activation by BPA and MBP; both chemicals targeted several molecular pathways constituting biomarkers for calcific aortic valve disease, including extracellular matrix alteration.	Brown *et al* (2019)[10]

Abbreviations: ERE, estrogen response element; MBP, metabolite bisphenol A; PFOS, perfluorooctane sulfonate; miRNA: microRNA.

Supplementary Table 9 **The cardiac developmental toxicity of bisphenol A (BPA) assessed in *in vitro* models**

Number	Experimental subject	Exposure mode	Results	Reference
1	hiPSC-CMs	During the differentiation protocol, BPA was administered at 0.01, 0.1, and 1 μmol/L. The exposure regimen, initiated at the pluripotency stage (day 0), involved treatment every second day.	BPA exposure impaired the contractility of human fetal cardiomyocytes, along with an increase in extracellular matrix protein deposition.	Lamberto *et al* (2023)[28]
2	R1 ES cells differentiation into cardiomyocytes	The exposure regimen from day 0 to 10 comprised individual treatments with 10 ng/mL of BPA, BPS, or BPAF, and co-treatments with a 1：1：1 mixture of the three compounds at two total concentrations (1 and 10 ng/mL).	Combined exposure to BPA, BPS, and BPAF disrupts cardiomyocyte differentiation in an additive manner and concurrently promotes the deposition of type Ⅰ and Ⅲ collagen at low concentrations.	Zhou *et al* (2021)[29]
3	H9c2 cells	A treatment window encompassing the terminal phase of differentiation (days 4–5) was used to expose cardiomyocyte differentiating cells to BPA at 10, 30, or 100 μmol/L.	BPA adversely affects the process of cardiomyocyte differentiation.	Escarda *et al* (2021)[9]
4	ESCs differentiation into myocardial cells	Throughout the 14-day differentiation period (days 0–14), ESCs were administered 10 ng/mL BPA, 100 ng/mL PFOS, or a mixture of both (10 ng/mL BPA + 100 ng/mL PFOS).	Co-exposure to PFOS and BPA induces mitochondrial damage, accompanied by cardiomyocyte enlargement.	Zhou *et al* (2020)[8]
5	mESCs differentiation into cardiomyocytes	Treatment of mESCs with P4 (10^{-8} mol/L), OP (10^{-6} mol/L), and BPA (10^{-7} mol/L) was initiated on day 2 post-attachment and continued for 10 days.	OP and BPA impair the cardiomyocyte differentiation of mESCs in a manner analogous to progesterone.	Lee *et al* (2019)[30]
6	Neonatal cardiomyocytes were isolated from 1-day-old Sprague-Dawley rats.	Cardiomyocytes underwent a 15-min treatment with BPA-supplemented media at final concentrations of 10^{-9} to 10^{-4} mol/L.	By disrupting intracellular calcium homeostasis, acute BPA exposure can adversely affect contractile performance and promote electrical alternans.	Ramadan *et al* (2018)[31]

Abbreviations: BPAF, bisphenol AF; BPS, bisphenol S; ESCs, embryonic stem cells; hiPSC-CMs, human induced pluripotent stem cells-derived cardiomyocytes; mESCs, mouse embryonic stem cells; OP, octyl-phenol; P4, progesterone; PFOS, perfluorooctane sulfonate.

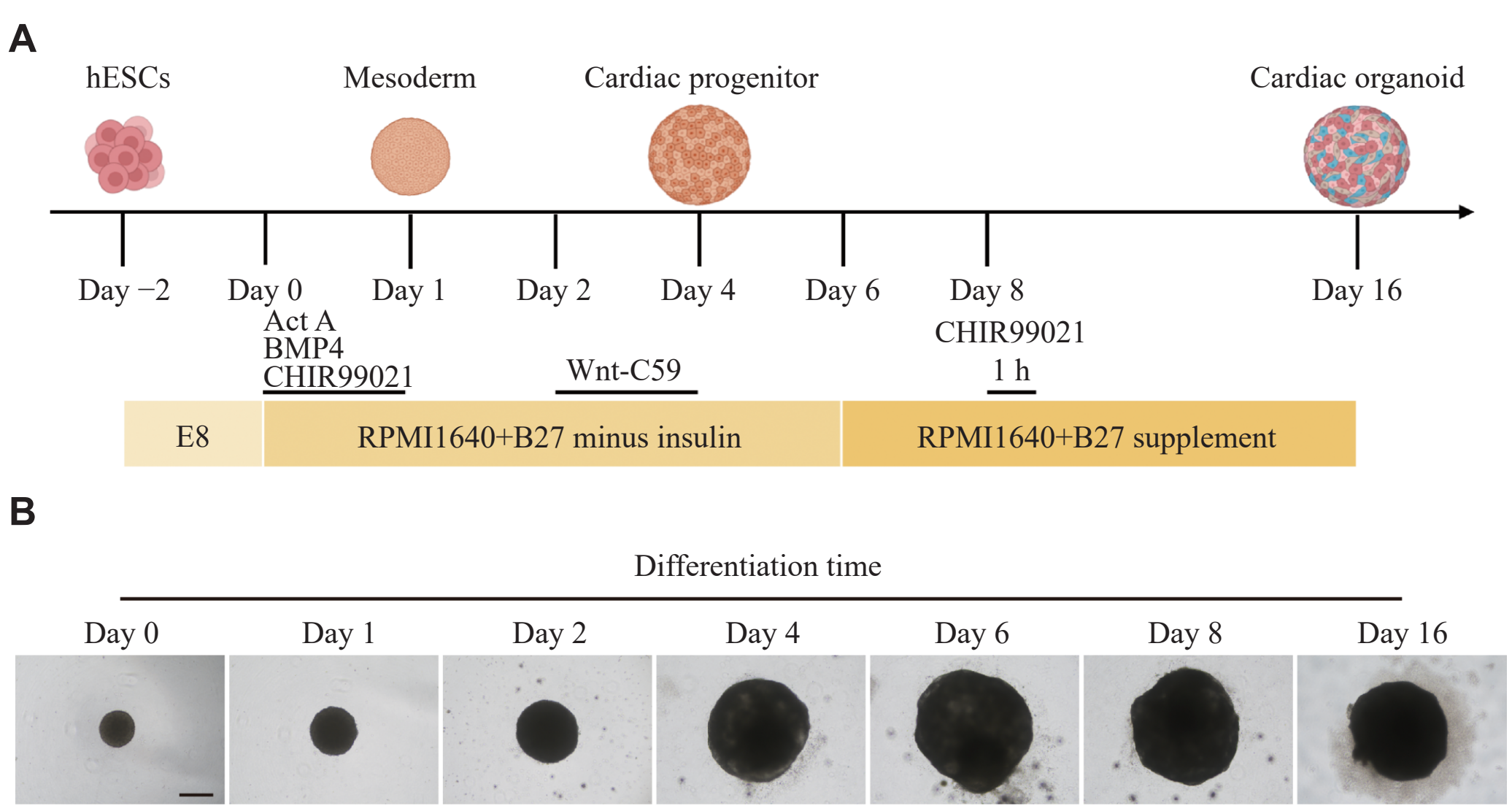

Supplementary Fig. 1 **Differentiation of human embryonic stem cells into human cardiac organoids (hCOs).** A: Schematic diagram of the differentiation protocol from human embryonic stem cells into hCOs. B: Brightfield images of hCOs at multiple time points during differentiation. Scale bar: 500 μm.

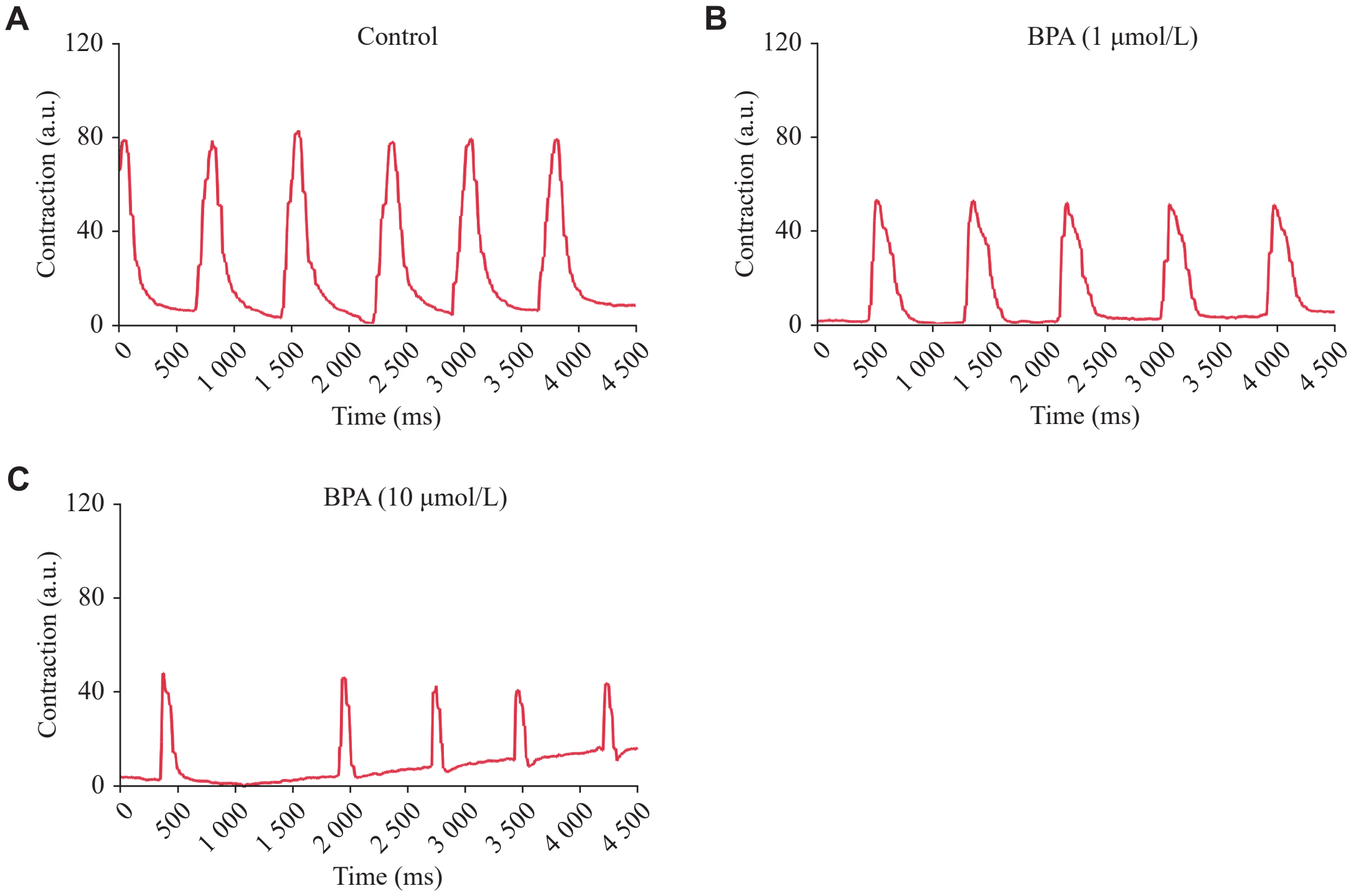

Supplementary Fig. 2 **Representative contractile images of human cardiac organoids under bisphenol A (BPA) exposure.** A: Control. B: 1 μmol/L BPA-treated. C: 10 μmol/L BPA-treated.

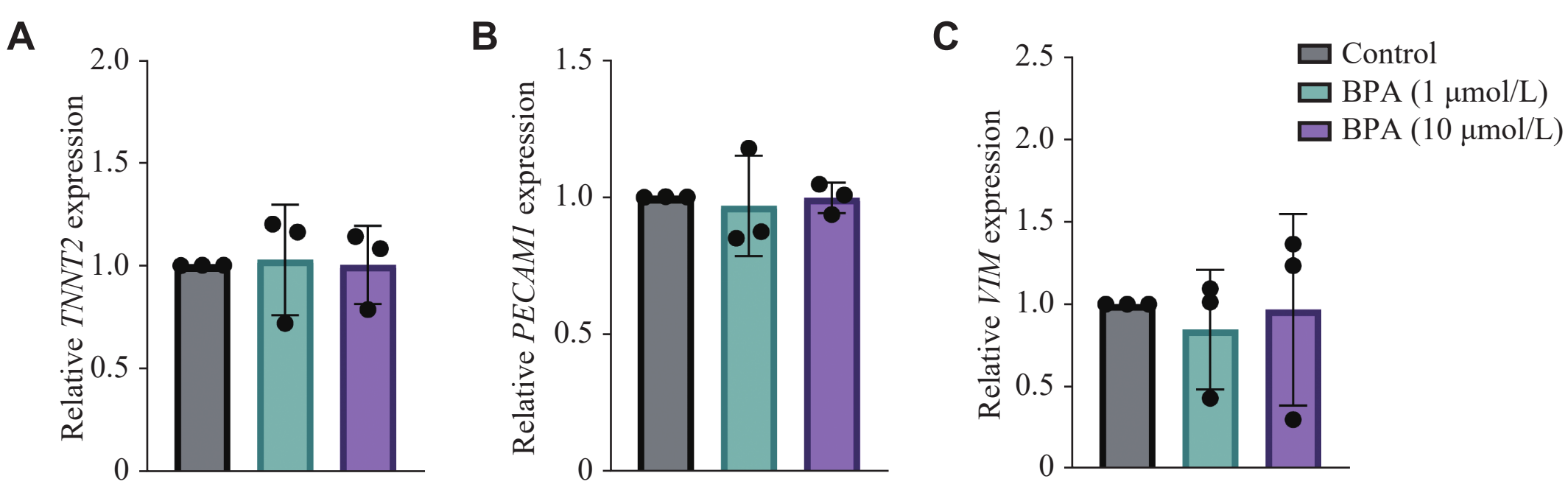

Supplementary Fig. 3 **BPA exposure did not alter the expression of key lineage markers during human cardiac organoid differentiation.** A–C: Relative mRNA expression of markers for major cardiac lineages (*TNNT2*, *PECAM1*, *VIM*) as determined by reverse transcription-quantitative PCR. Results are presented as mean ± standard deviation from three independent replicates and analyzed by one-way analysis of variance. Post hoc analysis was performed using Tukey's honestly significant difference test. Non-significant differences are not marked.

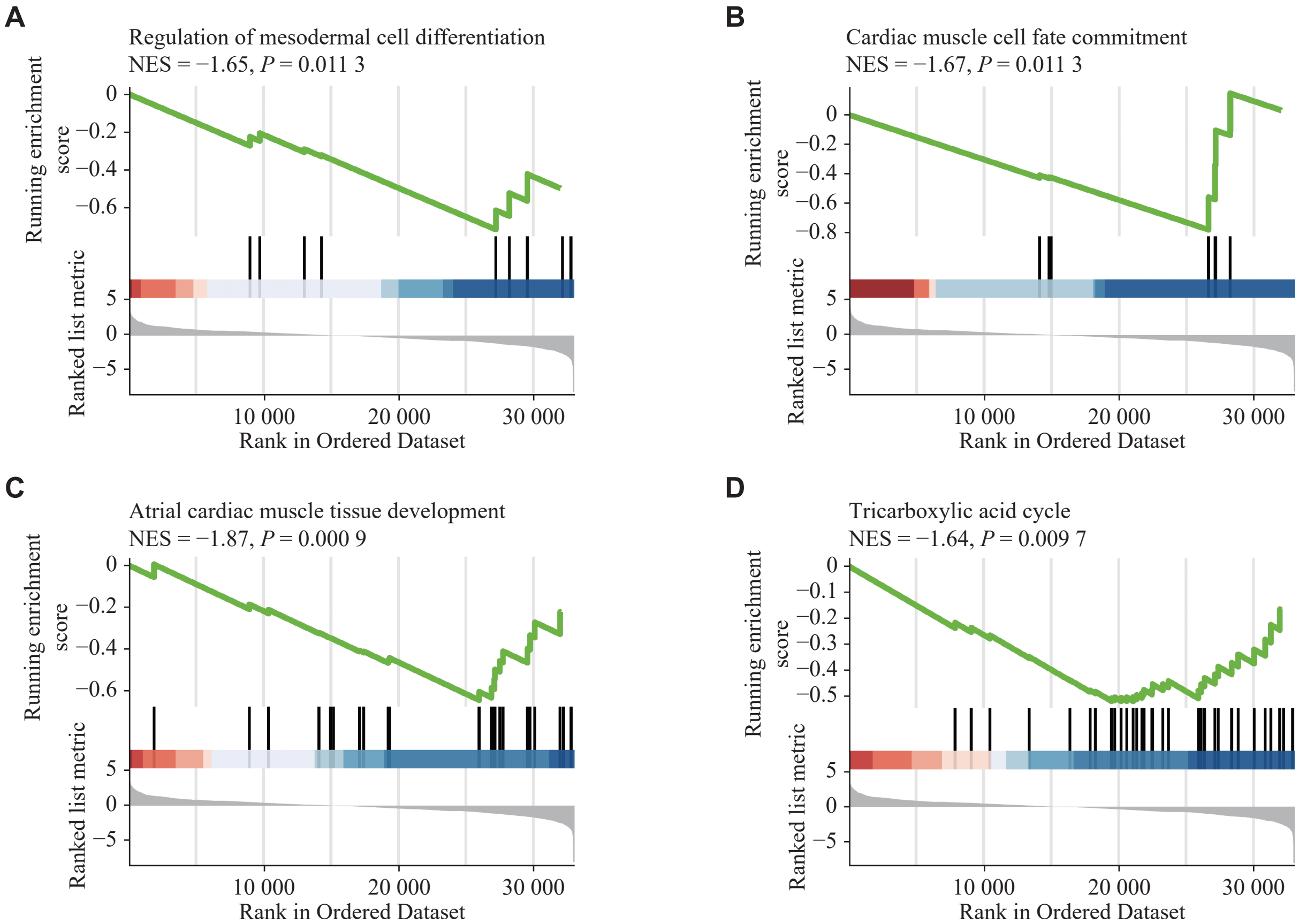

Supplementary Fig. 4 **Gene set enrichment analysis (GSEA) revealed the suppression of key biological processes by bisphenol A exposure.** A–D: GSEA plots showing significant suppression of regulation of mesodermal cell differentiation (A; normalized enrichment score (NES) = −1.65, P = 0.011 3), cardiac muscle cell fate commitment (B; NES = −1.67, P = 0.011 3), atrial cardiac muscle tissue development (C; NES = −1.87, P = 0.000 9), and tricarboxylic acid cycle (D; NES = −1.64, P = 0.009 7).

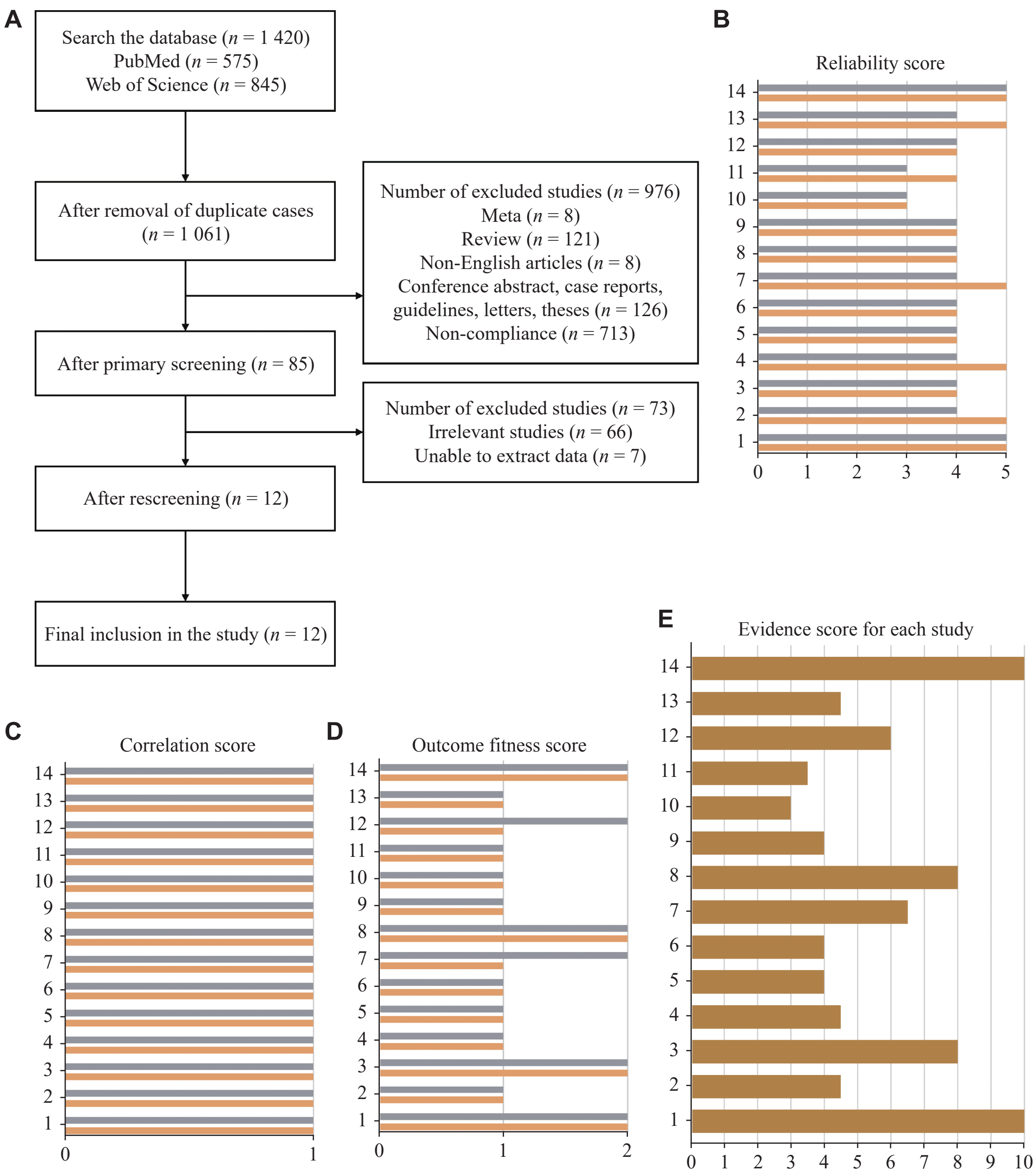

Supplementary Fig. 5 **Scoring was performed based on the Targeted Risk Assessment of Environmental Chemicals (TRAEC) criteria.** A: The flowchart illustrates the step-by-step process of identifying and selecting relevant studies for the systematic review. A total of 1 420 records were retrieved from two electronic databases (PubMed, *n* = 575; Web of Science, *n* = 845). After removing 359 duplicate records, 1 061 unique articles remained for the primary screening of titles and abstracts. This step led to the exclusion of 976 records for reasons including meta-analyses, reviews, non-English articles, conference abstracts, and irrelevance. The remaining 85 articles underwent full-text rescreening, resulting in the exclusion of 73 additional studies (66 were irrelevant to the research focus and 7 lacked extractable data). Ultimately, 12 studies met all the inclusion criteria and were selected for final analysis in the review. B: Distribution of reliability scores for included studies. C: Correlation scores reflecting the association strength between bisphenol A exposure and cardiac developmental outcomes. D: Outcome fitness scores across evaluated studies. E: Integrated evidence scores for individual studies. For B–E, the grey band represents the first researcher, and the orange band represents the second researcher. The x-axis represents score values, while the y-axis indicates the number of studies.

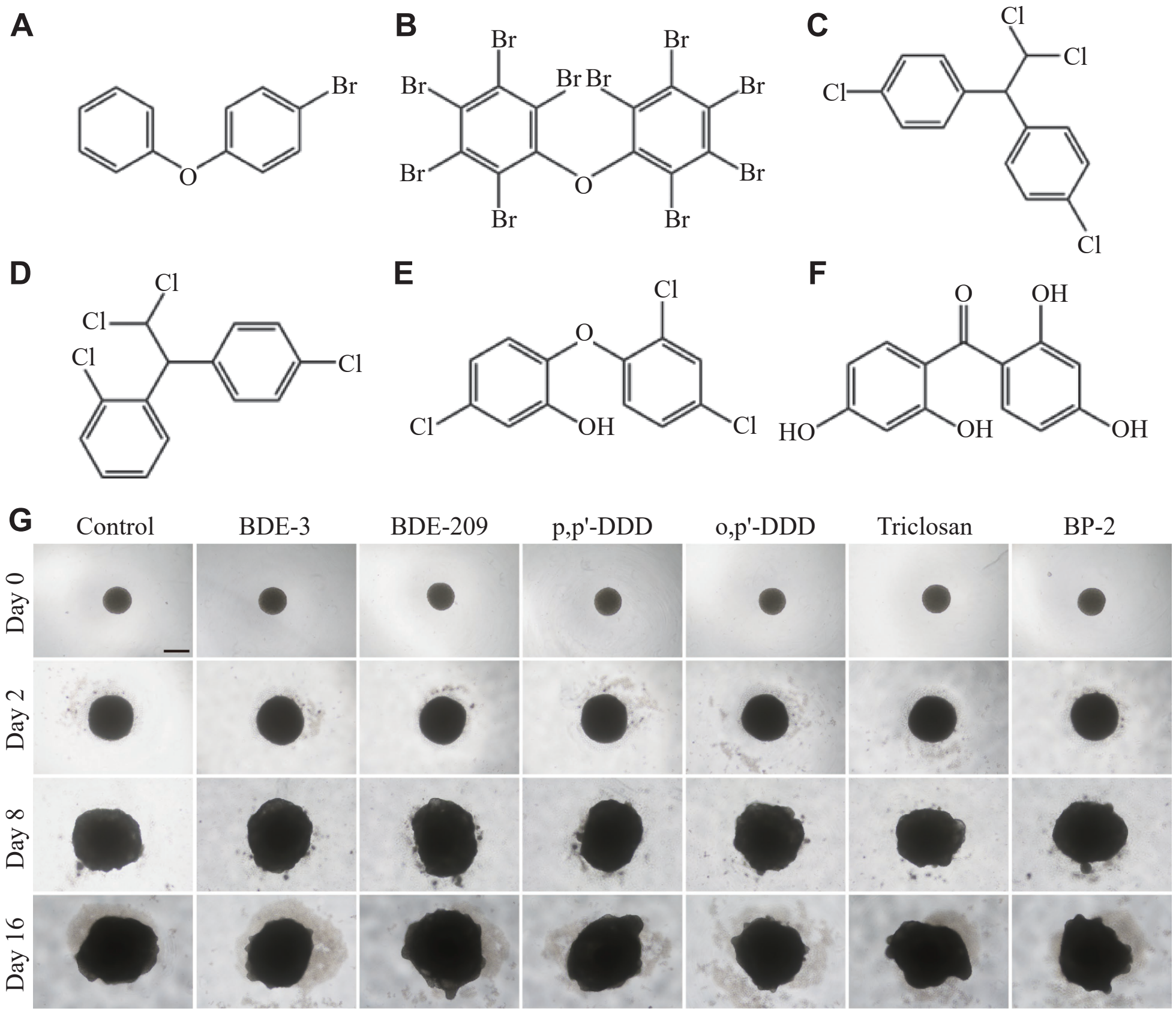

Supplementary Fig. 6 **Chemical structures of selected endocrine-disrupting chemicals (EDCs) and their effects on human cardiac organoid (hCO) morphology.** A–F: Chemical structures of BDE-3 ($C_{12}H_9BrO$) (A), BDE-209 ($C_{12}Br_{10}O$) (B), p,p'-DDD ($C_{14}H_{10}Cl_4$) (C), o,p'-DDD ($C_{14}H_{10}Cl_4$) (D), Triclosan ($C_{12}H_7Cl_3O_2$) (E), and BP-2 ($C_{13}H_{10}O_5$) (F). G: Bright-field images showing hCOs treated with various EDCs at selected time points. Scale bar: 500 µm.
